# Supplementary material for: Overexpression of CDCA2 in Diffuse Large B-Cell Lymphoma Promotes Cell Proliferation and Bortezomib Sensitivity
Source: Int J Mol Sci. 2025 Jun 11;26(12):5596. doi: 10.3390/ijms26125596 (PMC12193023; doi:10.3390/ijms26125596)
Supplement: Supplementary file 1 [file ijms-26-05596-s001.zip › Supplementary_materials_revised.pdf]

Supplementary material for:

Overexpression of *CDCA2* in diffuse large B-cell lymphoma promotes cell proliferation and bortezomib sensitivity

Content:

- **Supplementary Table S1.** Patient characteristics of the REMoDL-B data set.
- **Supplementary Table S2.** Prognostic genes with interaction to treatment arm.
- **Supplementary Table S3.** Multivariate Cox proportional hazards regression analysis.
- **Supplementary Table S4.** Multivariate Cox proportional hazards regression analysis.
- **Supplementary Figure S1.** CDCA2 expression levels vs. clinical and molecular subgroups.
- **Supplementary Figure S2.** Indel distribution of stable and transient CRISPR-edited cells.
- **Supplementary Figure S3.** Functional studies comparing parental wildtype and SCR control cells.
- **Supplementary Figure S4.** Gene set enrichment analysis of *CDCA2-KO* vs. SCR.
- **Supplementary Figure S5.** Flow cytometry analyses of SCR and *CDCA2-KO* cells.
- **Supplementary Figure S6.** Indel analysis of xenograft tumors.
- **Supplementary Figure S7.** IHC analysis of xenograft tumors.
- **Supplementary Figure S8.** Flow cytometry analysis of apoptosis in of SCR and *CDCA2-KO* cells.
- **Supplementary Figure S9.** Carfilzomib dose-response analysis.

Supplementary Table S1. Patient characteristics of the REMoDL-B data set.

	R-CHOP (N=469)	RB-CHOP (N=459)	Overall (N=928)
**Age**			
Mean (SD)	63.1 (12.2)	61.3 (12.6)	62.2 (12.4)
Median [Min, Max]	65.9 [24.1, 86.1]	63.1 [20.8, 84.5]	64.6 [20.8, 86.1]
Missing	0 (0%)	1 (0.2%)	1 (0.1%)
**Gender**			
Female	205 (43.7%)	206 (44.9%)	411 (44.3%)
Male	264 (56.3%)	253 (55.1%)	517 (55.7%)
**ECOG**			
0	256 (54.6%)	222 (48.4%)	478 (51.5%)
1	145 (30.9%)	168 (36.6%)	313 (33.7%)
2	52 (11.1%)	53 (11.5%)	105 (11.3%)
Missing	16 (3.4%)	16 (3.5%)	32 (3.4%)
**Stage**			
Stage I	13 (2.8%)	14 (3.1%)	27 (2.9%)
Stage II	133 (28.4%)	126 (27.5%)	259 (27.9%)
Stage III	129 (27.5%)	154 (33.6%)	283 (30.5%)
Stage IV	192 (40.9%)	163 (35.5%)	355 (38.3%)
Missing	2 (0.4%)	2 (0.4%)	4 (0.4%)
**IPI**			
High	82 (17.5%)	83 (18.1%)	165 (17.8%)
Intermediate	261 (55.7%)	256 (55.8%)	517 (55.7%)
Low	126 (26.9%)	120 (26.1%)	246 (26.5%)
**ABC/GCB**			
ABC	121 (25.8%)	123 (26.8%)	244 (26.3%)
GCB	240 (51.2%)	235 (51.2%)	475 (51.2%)
Unc	98 (20.9%)	101 (22.0%)	199 (21.4%)
Fail	10 (2.1%)	0 (0%)	10 (1.1%)
**MYC/BCL2 status**			
double-expressor	67 (14.3%)	59 (12.9%)	126 (13.6%)
non-double-expressor	114 (24.3%)	115 (25.1%)	229 (24.7%)
Missing	288 (61.4%)	285 (62.1%)	573 (61.7%)

Supplementary Table S2. Prognostic genes with interaction to treatment arm

	HR_{Arm}	P_{Arm}	HR_{Gene}	P_{Gene}	HR_{Int}	P_{Int}	Biological Process*
RTN4	-0.030	0.931	-0.321	0.008	0.677	0.029	Blastocyte formation, apoptotic process
CDCA2	-0.145	0.921	0.369	0.022	-0.597	0.080	Cell cycle
FZD3	-0.064	0.921	0.482	0.001	-0.623	0.080	Wnt signaling
PPIL3	0.014	0.964	0.423	0.001	-0.583	0.080	mRNA splicing, protein folding
TAF6	-0.078	0.921	0.282	0.029	-0.541	0.080	RNA polymerase II transcription
KLKB1	-0.126	0.921	-0.278	0.080	0.562	0.094	Blood coagulation, factor XII activation
LINC00472	-0.134	0.921	-0.287	0.055	0.538	0.094	-
PNPLA2	-0.029	0.931	0.303	0.022	-0.564	0.094	Triglyceride catabolic process
TMEM192	-0.016	0.959	-0.266	0.014	0.566	0.094	Transmembrane protein
ALG8	-0.027	0.936	0.443	0.001	-0.563	0.097	Protein N-linked glycosylation

Results from multiple Cox proportional hazards regression models with an interaction between gene expression and treatment arms. Table include genes with significant interaction ($p<0.1$). Columns show log (HR) for treatment arm, gene expression and interaction, with adjusted p-value. HR for the gene shows the effect in the R-CHOP group, with HR>0 suggestion worse survival for high expression. Arm, treatment arm; Gene, gene expression; Int, interaction; HR, hazard ratio; P, p value; *GO class.

Supplementary Table S3. Multivariate Cox proportional hazards regression analysis.

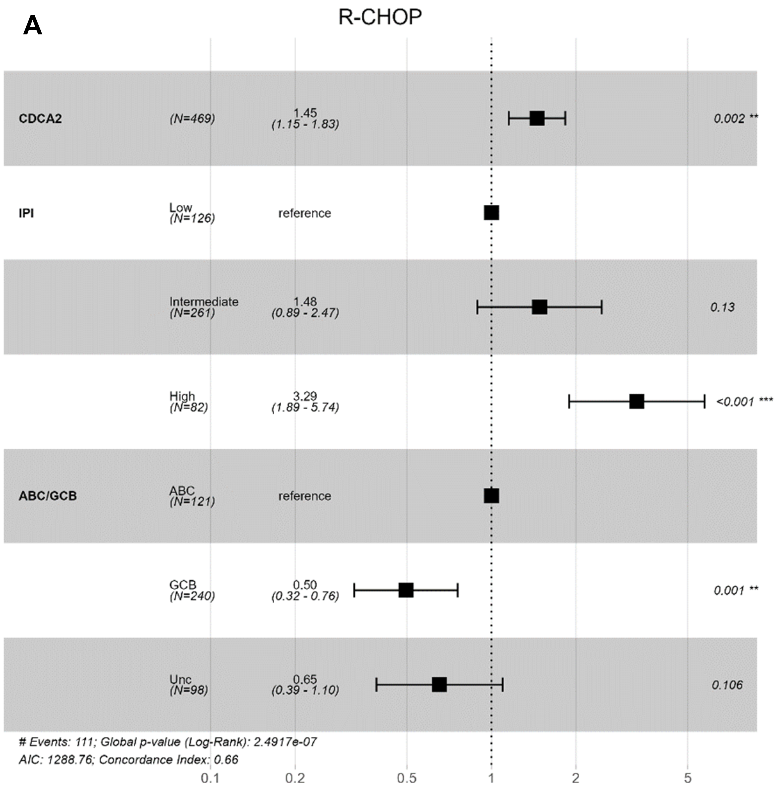

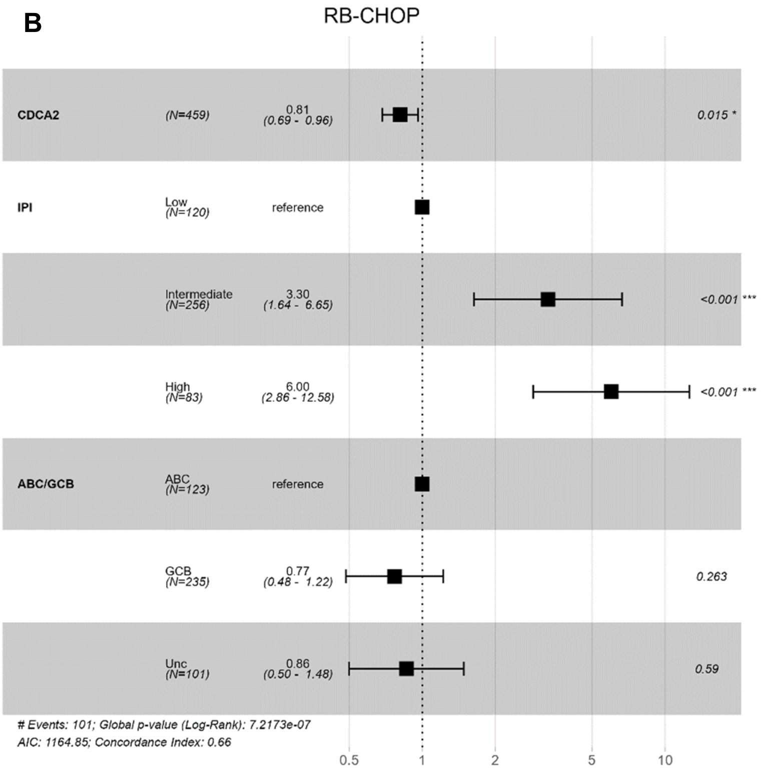

Supplementary Table S4. Multivariate Cox proportional hazards regression analysis in subset of patients with MYC/BCL2 expressor information.

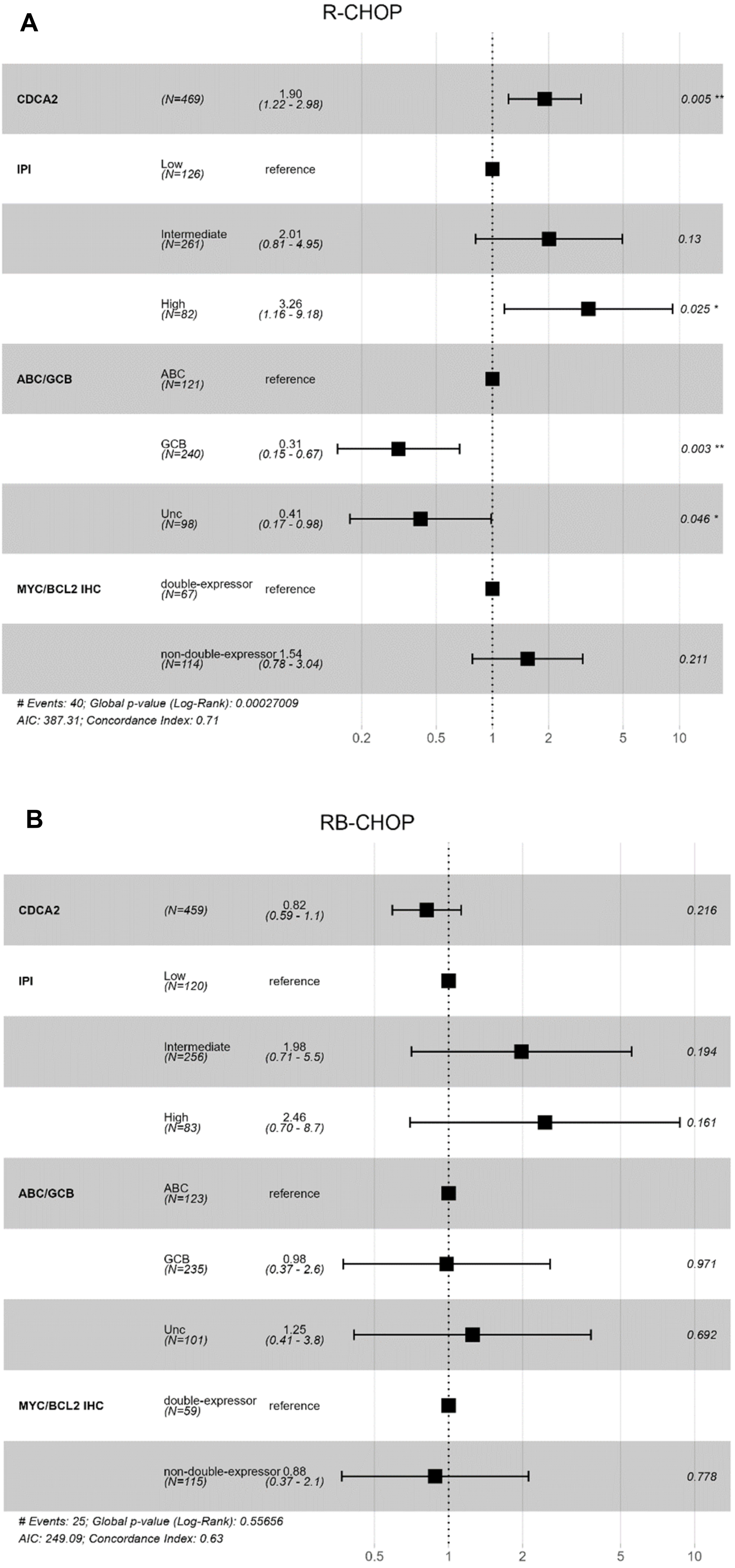

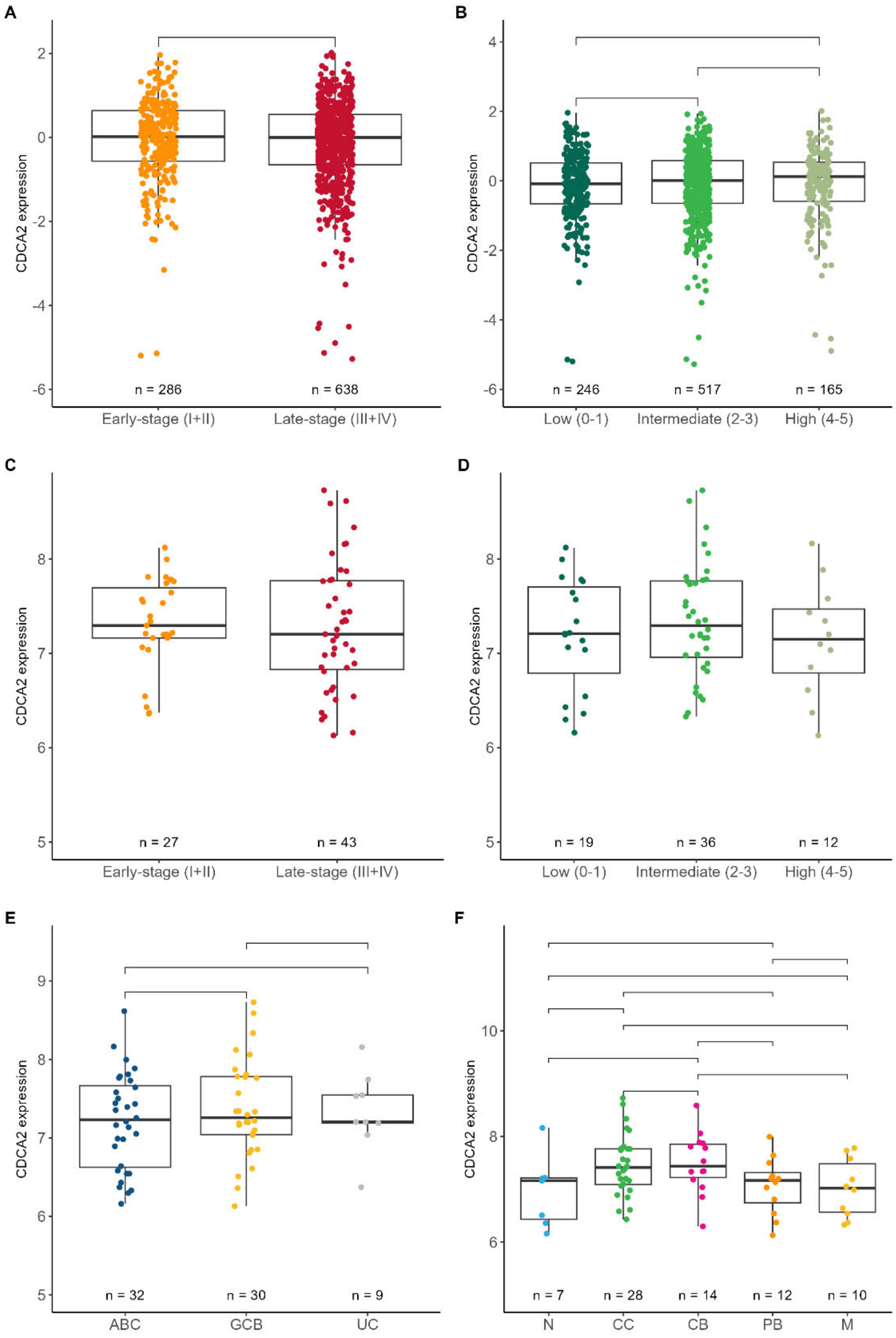

Supplementary Figure S1. *CDCA2* mRNA expression levels vs. clinical parameters. **A+B** Ann Arbor Stage and IPI score of patients in the REMoDL-B cohort. **C+D** Ann Arbor Stage and IPI score of patients from the local cohort. **E+F** ABC/GCB and BAGS subclassification of patients from the local cohort. Wilcoxon test. ABC, activated B-cell like; CB, centroblast-like; CC, centrocyte-like, GCB, germinal center B-cell-like; M, memory-like; N, naïve-like; PB, plasmablast-like; Unc, unclassified.

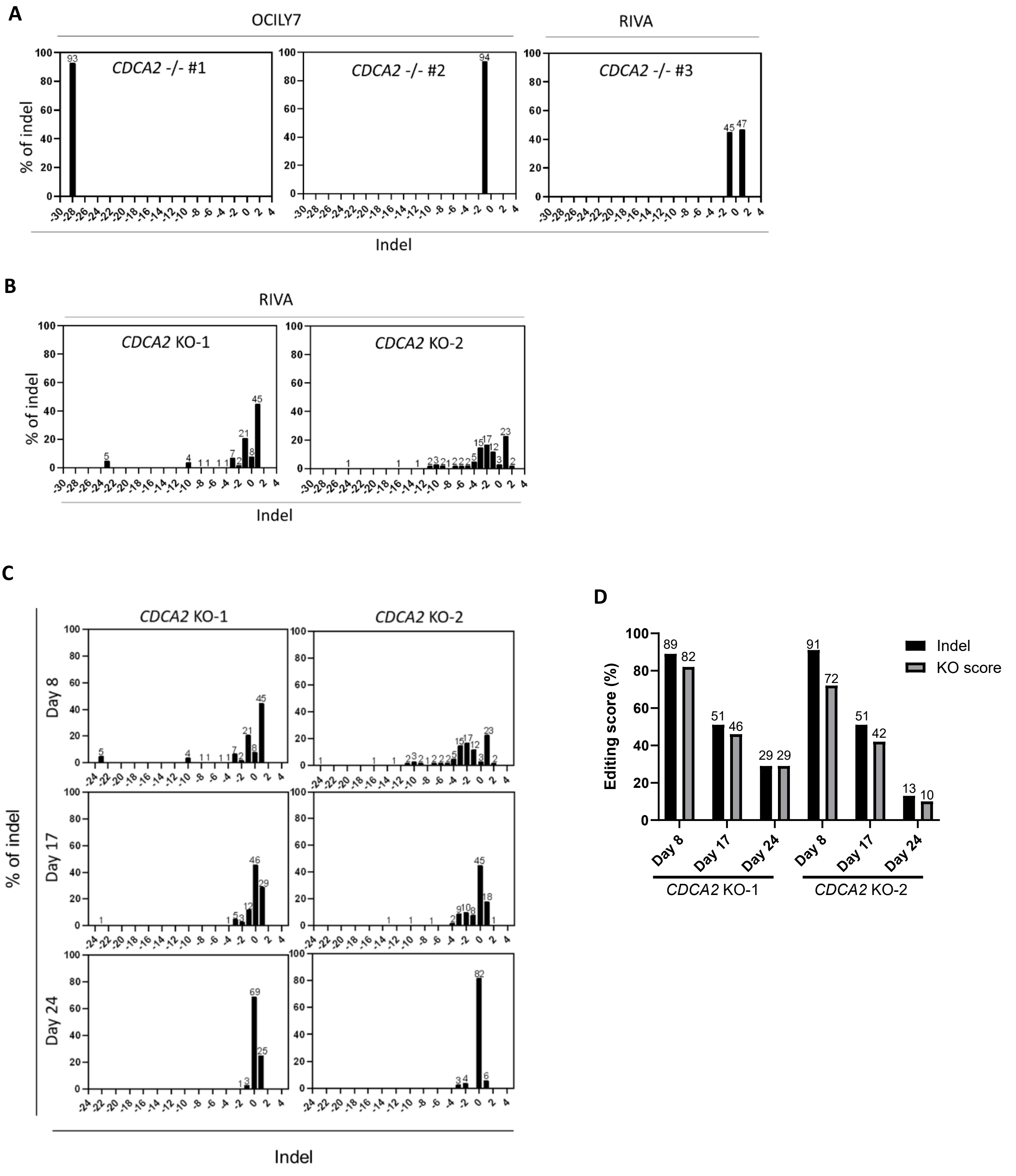

Supplementary Figure S2. Indel distribution of **A** lentiviral- and **B** RNP-based CRISPR-edited cells**. C** Indel distribution over time in RNP nucleofected RIVA cells. **D** Indel and knockout (KO) score for each gRNA at day 8, 17, and 24. Indels assessed by ICE analysis of Sanger sequencing output.

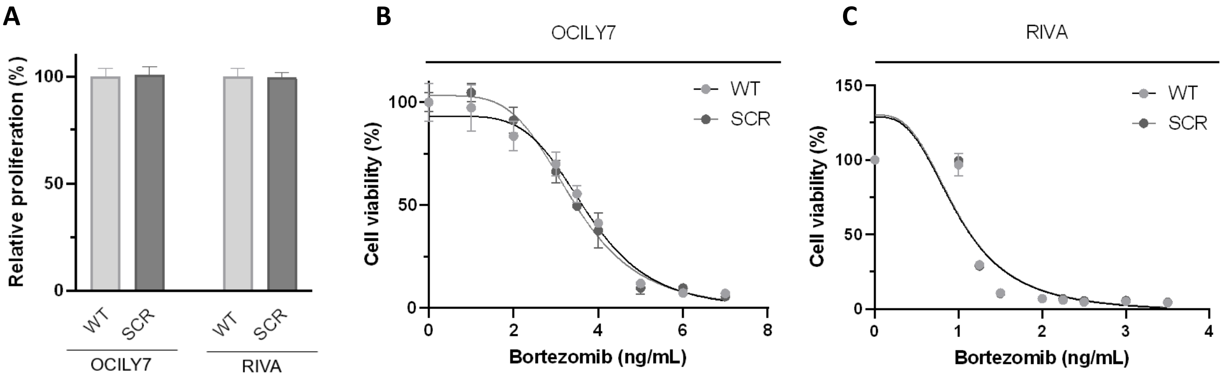

Supplementary Figure S3. Functional studies comparing parental wildtype and SCR control cells. **A** Proliferation of wildtype (WT) and scramble (SCR) control cells examined by MTS and presented relative to WT. **B+C** Bortezomib dose response analysis of WT and SCR control OCILY7 and RIVA cells, respectively. Cell viability was assessed by MTS and presented relative to the untreated control. Vales are displayed as mean ±SD.

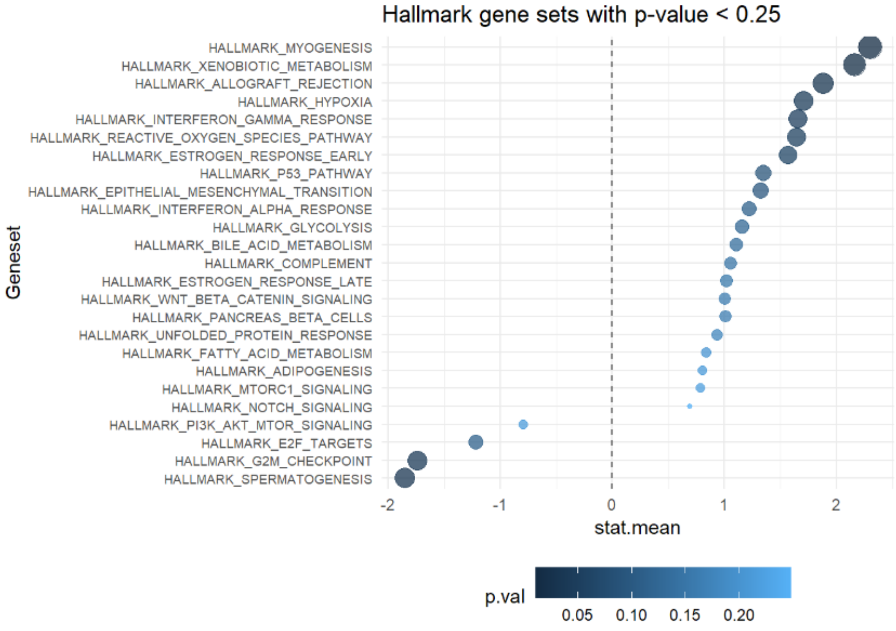

Supplementary Figure S4. Gene set enrichment analysis conducted on transcriptional profiles of OCILY7 *CDCA2-KO* (*CDCA2-/- #1* and *CDCA2-/- #2*) and SCR cells. GSEA was restricted to gene sets included in the Hallmark collection from the Molecular Signature Database.

A

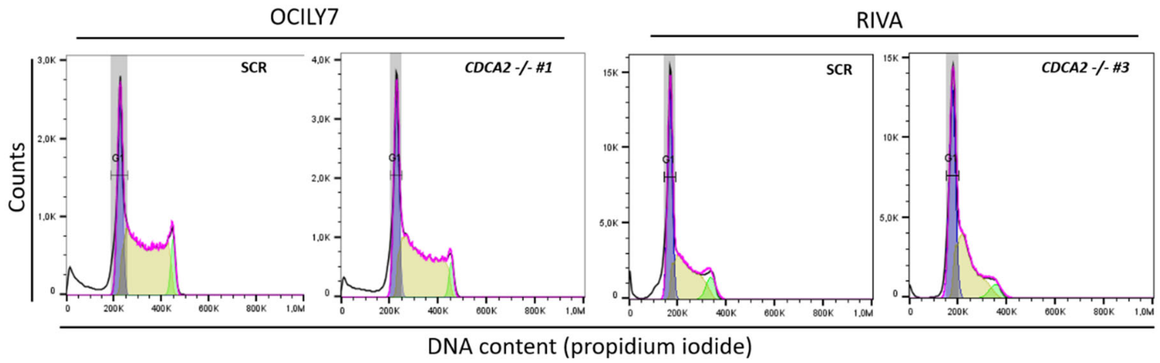

	Sample	% cells in G1	% cells in S	% cells in G2
OCILY7	SCR	24.1 [±1.1]	55 [±1.8]	5.1 [±0.8]
	CDCA2-/- #1	30.1 [±1.2]	52 [±1.3]	4.3 [±0.2]
RIVA	SCR	41.2 [±2.1]	40.4 [±0.9]	10.8 [±2.1]
	CDCA2-/- #3	45.8 [±1.2]	42.3 [±1.6]	7.4 [±1.6]

B

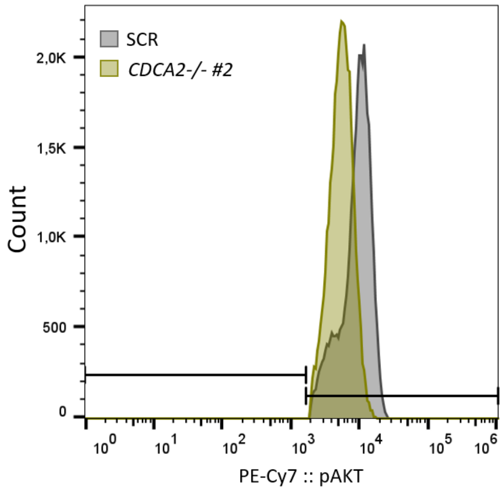

Supplementary Figure S5. Flow cytometry analyses of *CDCA2-KO* cells. **A** Cell cycle analysis by flow cytometry using propidium iodide. Cell cycle phases defined using univariate cell cycle model with Watson-Pragmatic statistics in FlowJo. Mean percentage of cells in respective phases presented in table, [± standard deviation]. **B** Phosphoflow analysis of AKT Ser473 (pAKT). Gating strategy included live cells (FSC/SSC), single cells, AKT positive cells, and lastly pAKT cells. Mean fluorescence intensity (MFI) of pAKT determined by FlowJo.

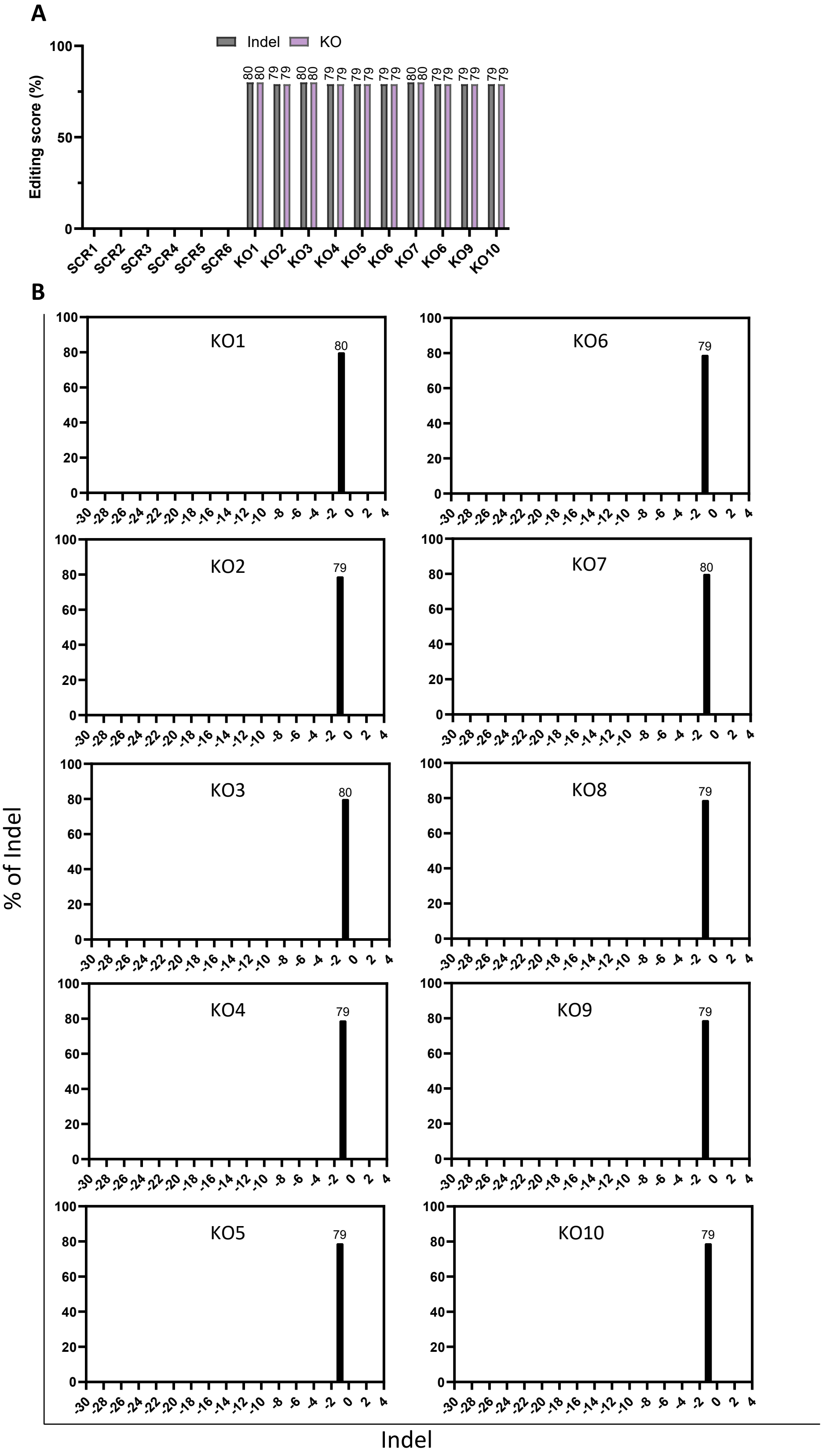

Supplementary Figure S6. Indel analysis of xenograft tumors at time of sacrifice. **A** Indel and knockout (KO) scores. **B** Indel distribution of each sample. SCR, scramble control. KO, *CDCA2-KO.*

A

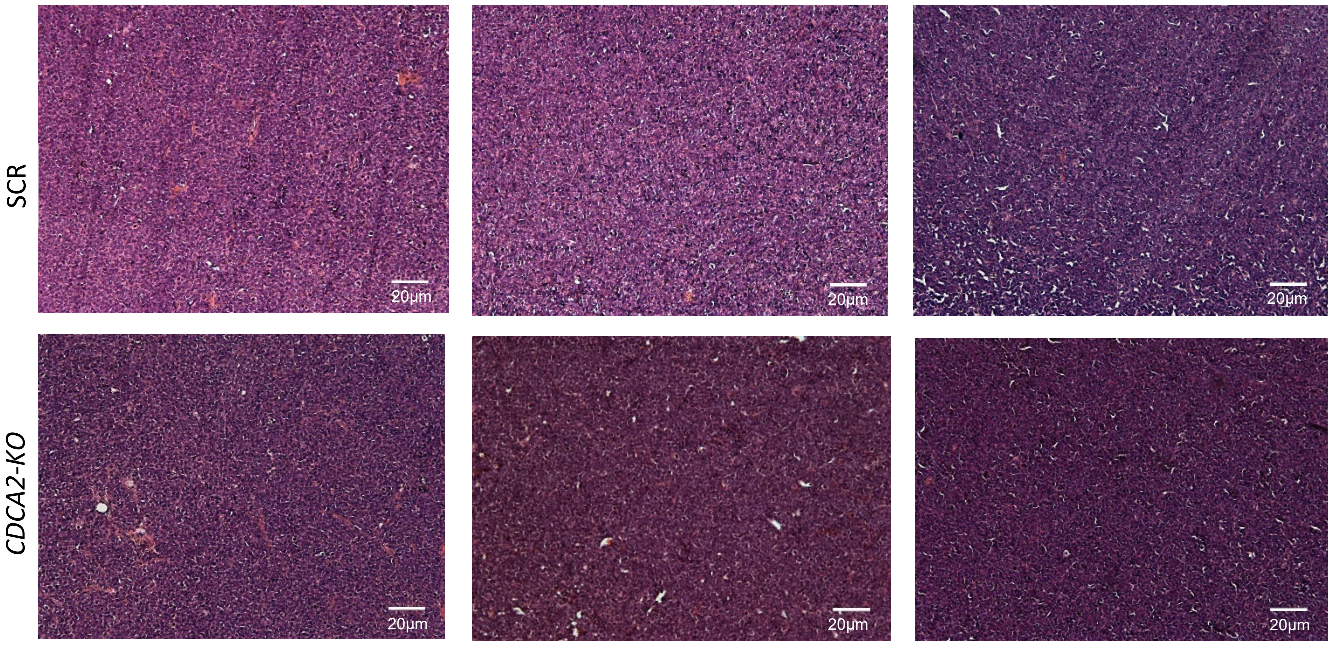

B

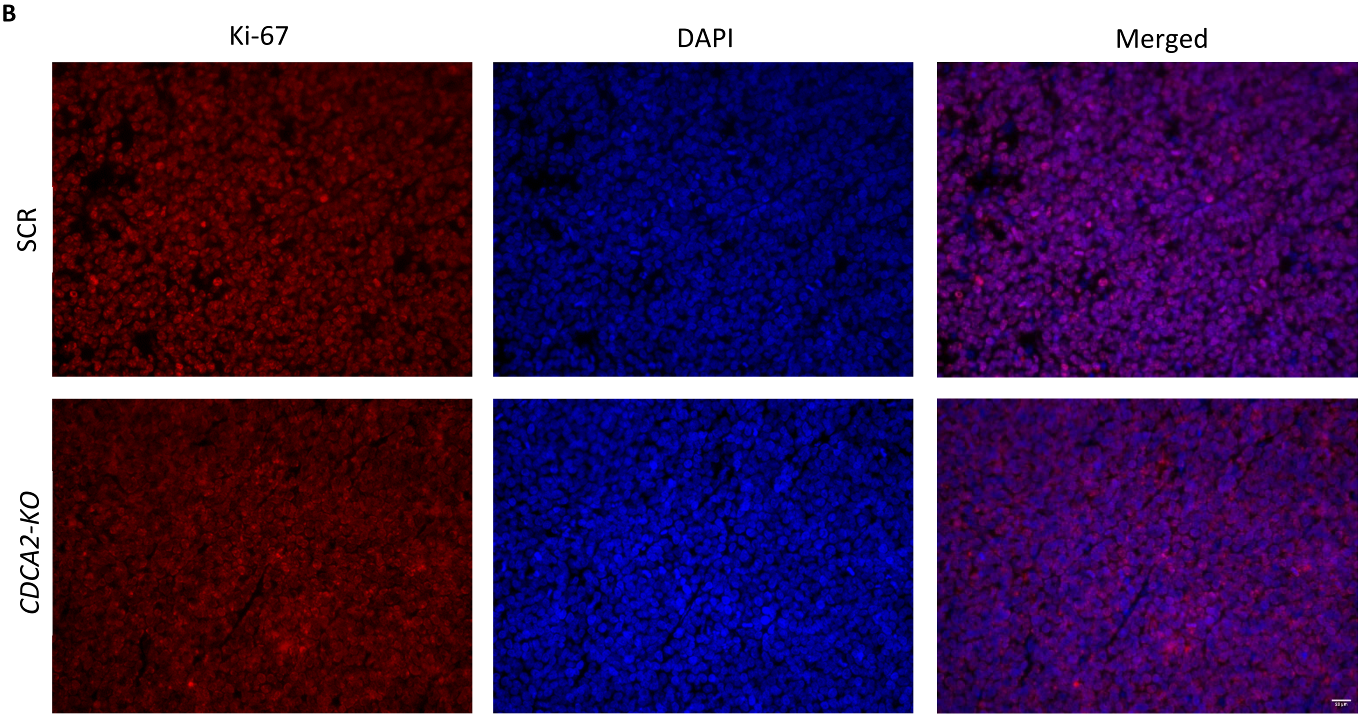

Supplementary Figure S7. IHC analysis of xenograft tumors using **A** HE and **B** Ki-67 and DAPI stain. Intensity was obtained by Fiji Image J. Ten images were obtained per sample. One image per group presented for visualization. Ki-67 intensity was normalized to DAPI intensity for each image and mean intensity for all 10 images was calculated prior to statistical testing. Scale bar is 20µm.

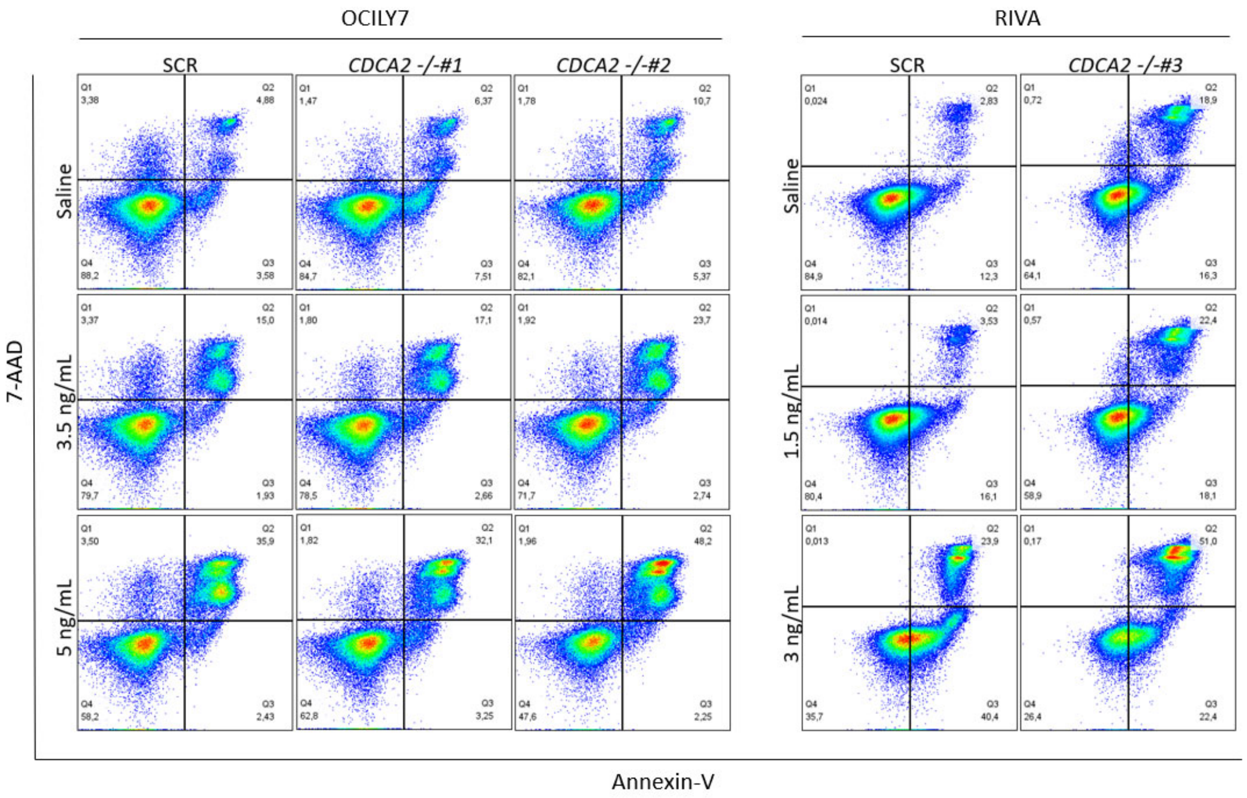

Supplementary Figure S8. Flow cytometry analysis of apoptosis in SCR and *CDCA2-KO* OCILY7 and RIVA cells treated with bortezomib.

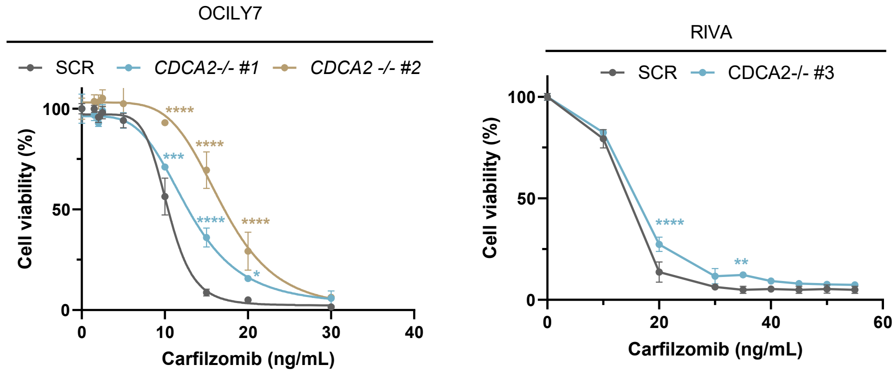

Supplementary Figure S9. Carfilzomib dose-response analysis in OCILY7 and RIVA cells. Cell viability determined by MTS and presented relative to the untreated control. -/- #cloneID represents monoclonal populations established by lentiviral transduction followed by single cell expansion. Values are presented as mean ±standard deviation. *P ≤ 0.05; **P ≤ 0.01; ***P ≤ 0.001; ****P ≤ 0.0001.
