# Supplementary material for: Overexpression of CDCA2 in Diffuse Large B-Cell Lymphoma Promotes Cell Proliferation and Bortezomib Sensitivity
Source: Int J Mol Sci. 2025 Jun 11;26(12):5596. doi: 10.3390/ijms26125596 (PMC12193023; doi:10.3390/ijms26125596)
Supplement: Supplementary file 1 [file ijms-26-05596-s001.zip › Supplementary_Methods_revised.pdf]

Supplementary Methods

REMoDL-B data set summary

This study was conducted on data from the REMoDL-B trial encompassing 928 de novo DLBCL patients which were randomized to receive R-CHOP (n=469) or RB-CHOP (n=459) [15,16]. In addition to clinical information and 5-year follow-up data, genome-wide gene expression data was available for all patients (Supplementary table 1). GEP was conducted on formalin-fixed paraffin-embedded tissue samples using Illumina whole genome cDNA-mediated annealing, selection, extension, and ligation assay (DASL) and used for molecular classification of patients to ABC, GCB, or Unc [15,16,38]. Moreover, immunohistochemical MYC/BCL2 double expressor status was available for a subset of 355 patients.

Analysis of REMoDL-B data

Gene expression data from the DASL platform was mapped to 19,267 genes. When multiple probes mapped to the same gene it was summarized by the average expression. To identify prognostic genes that interact with bortezomib treatment, we fitted Cox proportional hazards regressions with an interaction term between the treatment arm (R-CHOP or RB-CHOP) and single gene expression for all genes expressed on the DASL platform using overall survival as outcome. For the analysis, scaled-centered gene expressions were applied i.e., the expressed hazard ratio is for one standard deviation difference from the median gene expression. P-values were adjusted by Benjamini-Hocberg and significance level of 0.1 was chosen for the interaction term. Kaplan Meier survival analysis was conducted on DLBCL patients stratified by CDCA2 expression to high and low expression defined as above and below the median expression, respectively, calculated in the entire cohort irrespective of treatment arm. Multiple Cox proportional hazards regression analyses were conducted with interaction term between treatment and *CDCA2* adjusted for international prognostic index (IPI), ABC/GCB, and double-expressor status. Association between *CDCA2* expression levels quantified by the DASL array and clinical parameters including stage, IPI, and molecular subclasses including ABC/GCB, and double expressors were examined by pairwise comparisons for differences in distribution (median) using Wilcoxon test.

Analysis of local clinical data

Collection and analysis of clinical material was approved by the North Denmark Region Committee on Health Research Ethics. Diagnostic biopsies from 86 primary DLBCL patients were collected in accordance with the RetroGen Research protocol (N-20140099). Non-malignant tissue from healthy donors from approval N-

20080062MCH encompass 6 lymph nodes and 6 tonsils, and additional eight tonsils sorted by fluorescence-activated cell sorting (FACS) into following distinct B-cell subsets: naïve, centrocyte, centroblast, memory, and plasmablast as previously described [2]. For all samples, total RNA was labeled and hybridized to Affymetrix GeneChip Genome U133 Plus 2.0 Arrays [2]. Array data were background-corrected and normalized at gene level by robust multichip average (RMA) prior to analysis. Using gene expression profiles (GEP), DLBCL patients were classified into ABC/GCB subclasses and B-cell-associated gene signatures (BAGS) subtypes – a refined cell of origin classification system based on the normal B-cell differentiation hierarchy [2]. *CDCA2* expression levels from U133 array were examined for difference between DLBCL samples and normal samples, cell lines, and normal B-cell subsets, respectively, by pairwise comparison for difference in median using Wilcoxon test. Likewise, association between *CDCA2* mRNA levels and IPI, stage, ABC/GCB and BAGS subtypes were tested. BAGS classification was only performed in our local cohort as the classification system is only compatible with U133 array.

CRISPR/Cas9 knockout of *CDCA2*

Knockout was performed in DLBCL cell lines OCILY7 and RIVA. DLBCL cells were cultured in RPMI-1640 containing 10% fetal bovine serum (FBS) and 1% penicillin/streptomycin (P/S). HEK293T were maintained in DMEM, 10% FBS and 1% P/S. Cell lines were regularly authenticated by DNA barcoding and inspected for mycoplasma [37].

Guide RNAs (gRNAs) were designed using CRISPOR and CHOPCHOP [39,40]: gRNA1: 5'-ACAGTAACCGTAGAGCAATT-3' and gRNA2: 5'-GGTAATTCGGCATGCTTCTG-3'. A scrambled control (SCR) gRNA (5'-ACGGAGGCTAAGCGTCGCAA-3') without target sequences in the human genome was included.

For nucleofection, 3.2µg chemically modified gRNA (Synthego) and 6µg Cas9 protein (IDT #1081061) were incubated at 25°C for 15min. Cells (6×10^5) were harvested, washed, and resuspended in 20µL SG Cell Line Solution (Lonza #PBC3-00675) prior to addition of the ribonucleoprotein (RNP) complex of Cas9 and gRNA. Cells were nucleofected using the CM125 program at the 4D-nucleofector device (Lonza).

For the lentiviral delivery approach, the gRNA (Eurofins Genomics) was cloned into pLentiCRISPRv2 (Addgene p#52961), a gift from Feng Zhang [41]. Cloning was performed in a single-step digestion-ligation reaction using BsmBI (Thermo Scientific, FD0454) and T7 ligase (NEB, M0318S). Lentiviral vectors were produced by transfecting HEK293T cells with 1.24µg pMD2-G (Addgene #12259), 1µg pRSVRev (Addgene #12253), 4.3µg pMDLg/RRE (Addgene #12251), and 4.3µg cloned pLentiCRISPRv2 utilizing Lipofectamine 3000®. Viral supernatant was harvested 24- and 52-hours post-transfection and filtered (0.45µm). Yield was quantified by

measuring p24 capsid protein using HIV-1 p24 ELISA kit (Abcam, ab218268). DLBCL cells were transduced by seeding 3 x 10^5 cells in 1mL culture media in a 12-well plate with a multiplicity of infection of 1. Seventy-two hours post-transduction, successfully transduced DLBCL cells (GFP+) were FACS sorted into single cells in a 96-well plate (SH800, SONY). Single cells were cultured in media consisting of 50% 0.45µm-filtered conditioned media, 25% FBS, 24% RPMI-1640, and 1% P/S during expansion until cells were cultured in 6-well plates.

Primers for amplification of CRISPR knockout site: 5'-CCGAGGGCCTATTTCCCATGATTC-3' and 5'-TTCTCTAGGCACCGGATCAATTGC-3'. Following antibodies were used for Western Blotting: CDCA2 1:1,000 (Cell signaling #14976), β-actin 1:10,000 (Abcam, ab8227), goat anti-rabbit 1:10,000 (Abcam, ab6721).

Flow cytometry-based assays

Cells were seeded (0.5E6 cells in 1mL) in 12-well plates and incubated for 48h prior to harvest for analysis of cell cycle and apoptosis, and for 6h for AKT and Ser473 phosphorylation AKT (pAKT) analysis. For cell cycle and AKT/pAKT analysis, cells were washed twice in 1XPBS prior to ethanol fixation. Cells were dripped to 9mL of ice-cold 70% ethanol while vortexing at low speed and stored at -20°C for at least 24h. Ethanol-fixed cells were washed twice in 1XPBS. For cell cycle analysis, samples were resuspended in 435µL staining buffer (BD Pharmingen #554657) and incubated with RNase A (5µg, Qiagen 19101) at 37°C for 30min prior to addition of 15µL of propidium Iodide (BioLegend #421301) followed by 15 minutes of incubation at 4°C in the dark. For AKT/pAKT analysis, samples were resuspended in 95µL staining buffer and incubated with 5µL Akt (Cell signaling #8790) and 10µL phospho-Akt (Cell signaling #88106) for 1h at room temperature. For analysis of apoptosis, a positive apoptotic control was made by heating cells at 60°C for 15min. All samples were washed twice in 1xPBS, resuspended in Annexin-V binding buffer (BioLegend #640926), and added 5µL of Pacific Blue Annexin V and 7-AAD, respectively, followed by incubation at room temperature for 15min. Unstained controls were included and at least 100,000 events were analyzed by a SH800 flow cytometer (SONY). The experimental setups included technical triplicates in biological duplicates.

Dose response screens

Doxorubicin, vincristine, bortezomib, and carfilzomib were acquired from the pharmacy of Aalborg University Hospital. LY294002 was purchased from Selleckchem (S1105). Cyclophosphamide and prednisone are prodrugs that are converted into its active forms in the liver. Therefore, 4-hydroperoxy-

cyclophosphamide (Niomech #D-18864) and prednisolone (Selleckchem NSC-9900) were used. For combinatory drug studies of CHOP alone and in combination with bortezomib (B-CHOP), the composition of drugs was set as the clinical ratio of 83/5.5/0.16/11.1 and 0.18/83/5.5/0.22/11, respectively [15,42]. The individual drug compounds in 1µg/mL CHOP were; 2.82µM 4-hydroperoxy-cyclophosphamide, 0.1µM doxorubicin, 0.0018µM vincristine, and 0.27µM prednisolone and in 1µg/mL B-CHOP; 0.46µM bortezomib, 2.83µM 4-hydroperoxy-cyclophosphamide, 0.1µM vincristine, 0.31µM prednisolone.

Xenografts

Animal experimental procedures were performed in accordance with protocols approved by the Animal Experiments Inspectorate, Denmark. Female severe combined immunodeficiency (SCID) mice were housed in specific pathogen-free facility. A total of $1x10^7$ *CDCA2-KO* or SCR OCILY7 cells were resuspended in 100µL 1xPBS mixed with 100µL Matrigel (E1270, Sigma-Aldrich) and subcutaneous injected into the flanks of mice. Tumor size was measured every 3rd-4th day with a digital caliper. Mice were sacrificed when humane endpoint was reached (n_{SCR}=6, $n_{CDCA2\text{-}KO}$=10). Upon sacrifice, tumor tissue sections were snap frozen followed by DNA extraction and indel analysis. In addition, tissue sections were paraffin embedded followed by immunohistochemical analysis including HE, Ki-67 (14-5698-82, Invitrogen), and DAPI (Sigma-Aldrich). Images were obtained using an Axioplan 2 imaging microscope (Zeiss, Germany) with a fixed exposure time for all samples. Images were processed using Fiji Image J. Ten images were obtained for each sample, each normalized to DAPI intensity, prior to calculating the mean intensity for all 10 images per sample.
